# The TDP‐43/TP63 Positive Feedback Circuit Promotes Esophageal Squamous Cell Carcinoma Progression

**DOI:** 10.1002/advs.202402913

**Published:** 2024-07-18

**Authors:** Wenwen Li, Yanting Yang, Linying Huang, Xinyuan Yu, Teng Wang, Nasha Zhang, Ming Yang

**Affiliations:** ^1^ Shandong Provincial Key Laboratory of Radiation Oncology Cancer Research Center Shandong Cancer Hospital and Institute Shandong First Medical University and Shandong Academy of Medical Sciences Jinan Shandong 250117 China; ^2^ School of Life Sciences Shandong First Medical University and Shandong Academy of Medical Sciences Taian Shandong 271021 China; ^3^ Shandong University Cancer Center Jinan Shandong 250117 China; ^4^ Department of Radiation Oncology Shandong Cancer Hospital and Institute Jinan Shandong 250117 China; ^5^ Jiangsu Key Lab of Cancer Biomarkers Prevention and Treatment Collaborative Innovation Center for Cancer Personalized Medicine Nanjing Medical University Nanjing Jiangsu 211166 China

**Keywords:** esophageal squamous cell carcinoma, RNA‐binding protein, TDP‐43, TP63, transcription factor

## Abstract

Esophageal squamous cell carcinoma (ESCC) is one of the most prevalent malignancies with a 5‐year survival rate of only 15% in patients with advanced diseases. Tumor protein 63 (TP63), a master transcription factor (TF) in ESCC, cooperates with other TFs to regulate enhancers and/or promoters of target oncogenes, which in turn promotes tumorigenesis. TAR‐DNA‐binding protein‐43 (TDP‐43) is an RNA/DNA binding protein with elevated expression in several neoplasms. However, it remains unclear how TDP‐43 contributes to ESCC progression. In this study, *TDP‐43* is identified as a novel oncogene with markedly upregulated expression in ESCC tissues through profiling expression levels of one hundred and fifty canonical RNA binding protein (RBP) genes in multiple ESCC patient cohorts. Importantly, TDP‐43 boosted *TP63* expression via post‐transcriptionally stabilizing *TP63* mRNAs as a RBP and promoting *TP63* transcription as a TF binding to the *TP63* promoter in ESCC cells. In contrast, the master TF TP63 also bound to the *TDP‐43* promoter, accelerated *TDP‐43* transcription, and caused a noticeable increase in *TDP‐43* expression in ESCC cells. The findings highlight TDP‐43 as a viable therapeutic target for ESCC and uncover a hitherto unrecognized TDP‐43/TP63 circuit in cancer.

## Graphical abstract

*TDP‐43* is identified as a novel oncogene in esophageal squamous cell carcinoma. *TDP‐43* promotes *TP63* expression as an RNA binding protein and a transcription factor (TF). In contrast, the master TF TP63 also promotes *TDP‐43* transcription. The study highlights TDP‐43 as a viable therapeutic target and uncovers an unrecognized *TDP‐43/TP63* positive feedback pathway in cancers.

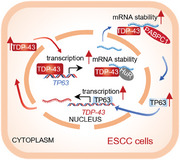

## 1 Introduction

Esophageal cancer is one of the most common malignancies and the sixth leading cause of cancer death globally. Its two histologic subtypes are esophageal adenocarcinoma (EAC) and esophageal squamous cell carcinoma (ESCC). In Eastern Asian, about 90% of esophageal cancer cases are ESCC.^[^ ^1^, ^2^ ^]^ The prognosis of ESCC is poor since most patients were usually diagnosed at advanced disease stages. Although systematic therapies (chemoradiotherapy,^[^ ^3^ ^]^ targeted therapy,^[^ ^4^ ^]^ and immune checkpoint inhibitor therapy^[^ ^5^ ^]^) have achieved great progress for disease control, the 5‐year survival rate for advanced ESCC is only 15%.^[^ ^2^, ^6^ ^]^ Lacking a fully understanding of pathological mechanisms contributing to the malignant progression of ESCC might be the main cause.

As a group of highly conserved proteins, RNA‐binding proteins (RBPs) are essential for post‐transcriptional regulation of gene expression.^[^ ^7^ ^]^ Forming RNA‐protein complexes (RNPs) is a frequent interaction between the conventional RBPs and particular RNA targets. Because the target RNAs are diverse and complicated, these RNPs play different roles during a variety of biological processes. It has been reported that multiple RBPs are dysregulated in cancers and involved in fine‐regulation of RNA metabolism of oncogenes and/or tumor suppressors.^[^ ^8^ ^]^ TAR‐DNA‐binding protein‐43 (TDP‐43, also known as TARDBP) is an RNA/DNA binding protein which belongs to the heterogeneous nuclear ribonucleoproteins (hnRNPs) family. On one hand, TDP‐43 can act as a canonical RBP to bind target RNAs. On the other hand, TDP‐43 functions as a transcription factor (TF) to bind specific DNA sequences. Consequently, TDP‐43 is essential for controlling RNA metabolism and gene transcription in human cells.^[^ ^9^, ^10^ ^]^ TDP‐43 has been reported as a pathological aggregating protein in several neurodegenerative diseases, including Alzheimer's disease, frontotemporal lobar degeneration, and amyotrophic lateral sclerosis, which are often associated with tau pathology.^[^ ^11^, ^12^ ^]^ Accumulated evidences indicated that TDP‐43 is also involved in development of malignancies, such as hepatocellular carcinoma (HCC), neuroblastoma and breast cancer, via regulating mRNA translation, RNA alternative splicing, RNA transport, RNA stability, and miRNA processing.^[^ ^13^, ^14^, ^15^ ^]^ For instance, by inhibiting the translation of GSK3β protein and activating the Wnt/β‐catenin pathway, TDP‐43 promotes growth and distant spread of HCC cells.^[^ ^16^ ^]^ However, it remains for the most part unclear how TDP‐43 contributes to ESCC progression.

As a member of the P53 family, tumor protein 63 (TP63) functions as a sequence‐specific DNA‐binding TF.^[^ ^17^, ^18^ ^]^ TP63 is involved in the regulation of cell metabolism, apoptosis, DNA repair, and proliferation.^[^ ^19^, ^20^ ^]^ During mammalian development, TP63 is essential for regenerative proliferation in limb, craniofacial, and epithelial development.^[^ ^21^ ^]^ In ESCC, TP63 acts as a master TF, cooperates with other TFs (DLX5, SOX2, and KLF5) to regulate enhancers and/or promoters of target oncogenes, and activates multiple oncogenic signaling pathways.^[^ ^22^, ^23^, ^24^ ^]^ Furthermore, TP63 also activates lncRNA transcription, such as *LINC01503*, by binding to its SCC‐specific super enhancer (SE).^[^ ^24^ ^]^ Highly expression of LINC01503 enhances migration, invasion, colony formation, and proliferation of ESCC cells.^[^ ^24^ ^]^ However, it is still largely unexplored how *TP63* is transcriptionally and post‐ transcriptionally regulated in ESCC.

In the present study, we profiled expression levels of 150 classical RBP genes in multiple ESCC patient cohorts and discovered TDP‐43 as an oncogene with significantly upregulated expression in ESCC tissues. *TDP‐43* promotes proliferation of ESCC cells ex vivo and in vivo. Importantly, TDP‐43 can significantly boost *TP63* expression via both stabilizing *TP63* mRNAs as a RBP at post‐transcriptional level and enhancing *TP63* transcription as a TF binding to the *TP63* promoter at transcriptional level in ESCC cells. In contrast, the TF TP63 attaches to the TDP‐43 promoter, speeds up transcription, and causes noticeably elevated TDP‐43 expression in ESCC. Our data identify a previously underappreciated TDP‐43/TP63 positive feedback circuit and illustrate that activation of the circuit promotes esophageal tumorigenesis.

## 2 Experimental Section

### 2.1 Cell Culture

The RPMI 1640 medium (Gibco, USA) supplemented with 10% fetal bovine serum (FBS, Gibco) was used for cultivating the KYSE‐450, KYSE‐510, KYSE‐30, KYSE‐180, KYSE‐520, and HEK293T cells. Prof. Dongxin Lin (Department of Etiology and Carcinogenesis, National Cancer Center/National Clinical Research Center/Cancer Hospital, Chinese Academy of Medical Sciences and Peking Union Medical College, Beijing, China) kindly contributed the human KYSE‐450, KYSE‐510, KYSE‐30, KYSE‐180 and KYSE‐520 ESCC cell lines. Dr. Yunshan Wang (Jinan Central Hospital, Shandong Province, China) generously contributed HEK293T cells. The cells were cultivated in an incubator with 5% CO_2_ at 37 °C, and their mycoplasma negative status was checked on a regular basis.

### 2.2 Overexpression, shRNA, and Mutant Constructs of TDP‐43

The pCDH‐CMV‐MCS‐EF1α‐Puro vector (Genewiz, Suzhou, China) was used to clone the full‐length *TDP‐43* cDNA (NM_007375.4). The resulting plasmid was given the name TDP‐43. HA‐tagged wild‐type (WT) full‐length *TDP‐43* cDNA was also cloned into pcDNA3.1. A negative control shRNA (shNC) and two *TDP‐43* shRNAs (shT43‐1 or shT43‐2) were synthesized and cloned into the pLKO.1 vector (Table S3, Supporting Information). After deleting the 105–169 nt, 193–252 nt, or the 105–169 nt plus 193–252 nt regions of wild‐type (WT) full‐length *TDP‐43* mRNA, three truncated TDP‐43 plasmids (ΔRRM1, ΔRRM2, or ΔRRM1&RRM2) with a HA‐tag were produced. To verify the orientation and integrity, all plasmids were sequenced.

### 2.3 Cell Transfection

Transfection of all plasmids was carried out using the jetPRIME reagent (Polyplus, USA). Small interference RNAs (siRNAs) of *TP63* (siTP63‐1 and siTP63‐2), *TDP‐43* (siT43‐1 and siT43‐2), *PABPC1* (siPABPC1‐1 and siPABPC1‐2), *HuR* (siHuR‐1 and siHuR‐2) as well as the negative control RNA (NC RNA) were products of Genepharma (Shanghai, China) (Table S3, Supporting Information). Small RNAs were transfected using the INTERFERin reagent (Polyplus, USA), as previously reported.^[^ ^25^, ^26^, ^27^ ^]^

### 2.4 Lentiviral Transduction

The lentivirus plasmid TDP‐43, shT43‐1, or shT43‐2 was co‐transfected into HEK293T cells along with the psPAX2 (Addgene, #12260) and pMD2.G (Addgene, #12259) plasmids as reported previously.^[^ ^25^, ^26^, ^27^ ^]^ Recombinant lentiviral particles in the viral supernatants were extracted 48 and 72 h after transfection. ESCC cells were infected with the viral particles of TDP‐43, shT43‐1, shT43‐2, NC, or shNC independently. Cells were then selected using blasticidin or puromycin at a concentration of 2 µg mL^−1^.

### 2.5 Quantitative Reverse Transcription PCR (RT‐qPCR)

Reverse transcribed total RNAs were used to determine the relative expression of candidate genes using the indicated primers (Table S2, Supporting Information) as reported previously.^[^ ^25^, ^26^, ^27^ ^]^

### 2.6 Western Blotting

Western blot was performed as described previously.^[^ ^25^, ^26^, ^27^, ^28^ ^]^ Using an SDS‐PAGE gel, total proteins were isolated and then transferred to a PVDF membrane (Millipore, ISEQ00010). Next, the indicated antibodies were incubated with the PVDF membrane (Table S1, Supporting Information). The ECL Western Blotting Substrate (Pierce, 32106) was used to visualize proteins.

### 2.7 Cell Proliferation and Colony Formation Assays

Stable *TDP‐43*‐knockdown (*TDP‐43*‐KD) or *TDP‐43*‐overexpression (*TDP‐43*‐OE) KYSE‐450 or KYSE‐510 cells were used to investigate the functions of *TDP‐43* in cell proliferation and colony formation as reported previously.^[^ ^28^, ^29^ ^]^

### 2.8 Wound Healing and Transwell Assays

As previously reported, transwell assays and wound healing assays were used to examine the effects of TDP‐43 on the migratory and invasion capacities of ESCC cells.^[^ ^25^, ^26^, ^27^ ^]^

### 2.9 Xenografts

In order to investigate the role of *TDP‐43* in vivo, 4 × 10^6^ *TDP‐43*‐KD KYSE‐450 cells, 4 × 10^6^ *TDP‐43*‐OE KYSE‐450 cells, or 4 × 10^6^ control cells were injected subcutaneously into the scapular area of 5 weeks old male BALB/c nude mice (Vital River Laboratory, Beijing, China) (*n* = 5 per group). Tumor volumes were assessed every two days as previously described.^[^ ^26^, ^27^ ^]^ All procedures involving mice were approved by the Ethics Committee of Shandong Hospital and Institute (SDTHEC2021012032).

### 2.10 RNAseq

NovaSeq6000 (Illumina, USA) was used to perform RNA‐seq of *TDP‐43*‐KD KYSE‐450 cells to identify the target genes of TDP‐43 in ESCC cells. Differentially expressed genes (|log2(Fold Change)|>2, *P* < 0.05) were subjected to the KEGG (Kyoto Encyclopedia of Genes and Genomes) pathway analyses.

### 2.11 Chromatin Immunoprecipitation Sequencing (ChIP‐seq) and ChIP‐qPCR

The ChIP assays were performed using anti‐TDP‐43 antibody, anti‐TP63 antibody, or the IgG control as described previously.^[^ ^25^, ^27^ ^]^ Utilizing the Illumina NovaSeq 6000 platform (Illumina, USA), immunoprecipitated DNA was sequenced. The ChIP‐qPCR assays were performed using the ChIP‐qPCR primers (Table S2, Supporting Information) as previously described.^[^ ^25^, ^27^, ^28^, ^29^ ^]^

### 2.12 RNA Immunoprecipitation Sequencing (RIP‐seq) and RIP‐qPCR

The Magna RIP RNA‐Binding Protein Immunoprecipitation Kit (Millipore, 17–700) with either the anti‐TDP‐43 antibody or the IgG control were used for RIP assays. The target RNPs were enriched with Dynabeads Protein G beads (Invitrogen). A magnet was used to immobilize the magnetic beads‐bound RNPs. After that, the beads were washed for six times. The immunoprecipitated RNAs were sequenced on an Illumina NovaSeq 6000 (Illumina, USA) or detected via RIP‐qPCR.

### 2.13 Dual Luciferase Reporter Gene Assays

The potential *TP63* promoter region (chr3: 189629603–189629853 bp) or the *TDP‐43* promoter region (chr1: 11025262–11025612 bp) was cloned into the pGL3‐Basic vector (Promega, Madison, WI), respectively. The *TDP‐43* reporter construct was designated as pGL3‐TDP‐43‐WT. The pGL3‐TDP‐43‐WT construct with two deleted TP63‐binding motifs were named as pGL3‐TDP‐43‐del‐a and pGL3‐TDP‐43‐del‐b. The *TP63* reporter construct was named as pGL3‐TP63‐WT. The construct with deleted TDP‐43‐binding motif was designated as pGL3‐TP63‐del. As a normalization control, the pRL‐SV40 plasmid was co‐transfected with these constructs into cells. Luciferase activities were measured with the Dual‐Luciferase Reporter Assay System (Promega).

### 2.14 mRNA Stability Assays

To determine the impacts of TDP‐43 as a RBP on *TP63* mRNA stability, ESCC cells were treated with 0.25 mg mL^−1^ actinomycin D and harvested at the indicated time points after drug treatment. qRT‐PCR was performed to examine *TP63* mRNA levels in ESCC cells.

### 2.15 Immunoprecipitation‐Mass Spectrometry (IP‐MS) and Co‐IP

To identify proteins interacting with TDP‐43 in ESCC cells, IP‐MS was carried out using the anti‐TDP‐43 antibody. The immunoprecipitated proteins were analyzed using liquid chromatography‐tandem mass spectrometry (LS‐MS/MS) (Hoogen Biotech Co., Shanghai, China) as described previously.^[^ ^30^ ^]^ Co‐IP was performed between TDP‐43 and HuR, or TDP‐43 and PABPC1 in ESCC cells. Western blotting was used to examine the proteins that were retrieved with a total of 1% of inputs used.^[^ ^28^, ^29^ ^]^

### 2.16 Immunofluorescence

The immunofluorescence assays were carried out as reported previously.^[^ ^25^, ^27^, ^30^ ^]^ TDP‐43, HuR, and PABPC1 proteins were detected with the indicated antibodies and coraLite488‐conjugated or coraLite594‐conjugated secondary antibodies, followed by washing with PBS and staining with 4,6‐diamidino‐2‐phenylindole (DAPI). Cells images were visualized and recorded with a Zeiss LSM800 confocal microscope (Zeiss, Germany).

### 2.17 Statistics

The Student's *t*‐test was used to determine the difference between the two groups. One‐way ANOVA was employed to compare data from multiple groups. The significance of expression associations between different genes was examined using Spearman's correlation analyses. Statistical significance was defined as a *p‐*value of <0.05. The SPSS software package (Version 16.0, SPSS Inc.) or GraphPad Prism (Version 8, GraphPad Software, Inc.) were used for all analyses.

## 3 Results

### 3.1 Elevated Expression of TDP‐43 in Malignant Tissues was Associated with Poor Prognosis of ESCC Patients

**Figure 1:**
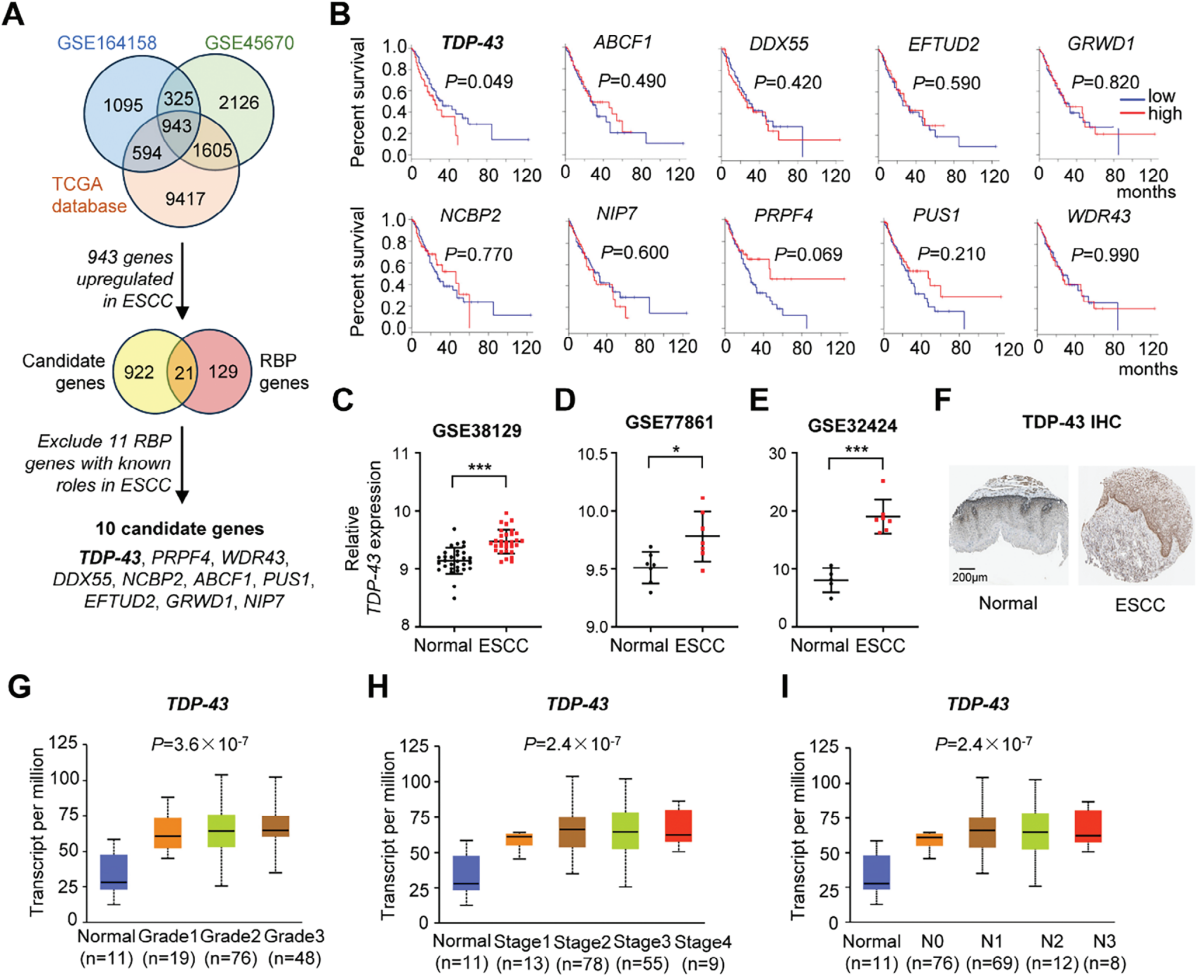
TDP‐43 is highly expressed in ESCC and associated with poor prognosis of patients. A) An illustration showing the RNA‐binding proteins (RBPs) that are up‐regulated in ESCC of the TCGA and GEO (GSE164158 and GSE45670) cohorts. B) Kaplan–Meier curves showed that high levels of *TDP‐43* were significantly associated with poor survival in the TCGA ESCA cohort. C–E) There were elevated *TDP‐43* expression in ESCC tissues compared to normal specimens in the other three cohorts (GSE38129, GSE77861, and GSE32424). F) Expression of TDP‐43 in ESCC and normal tissue was determined by IHC (proteinatlas.org). G–I) Differential *TDP‐43* expression in cancerous tissues of patients with esophageal carcinoma of different grades (G), clinical stages (H), and lymph nodal metastasis status (I) in the TCGA ESCA cohort. Data represent mean ± S.D., the *p‐*value was determined by a two‐tailed paired *t*‐test or unpaired Student's *t*‐test or one‐way ANOVA. Log‐rank test was used for survival comparison. ^*^ *p* < 0.05, ^***^ *p* < 0.001.

To explore the role of RBPs in ESCC progression, we examined the expression profiling in ESCC specimens of one hundred and fifty canonical RBP genes from TCGA and two other cohorts (GSE164158 and GSE45670) (**Figure** **1**A). There were twenty‐one RBP genes with significantly elevated expression in ESCC tissues compared to normal specimens in all three cohorts. Eleven RBP genes were excluded due to their known roles in ESCC and ten candidate genes were identified (Figure 1A). Among these ten RBP genes, only high levels of *TDP‐43* in ESCC tissues were remarkably associated with a shorten overall survival (OS) time in ESCC patients from the TCGA cohort (Figure 1B). Considering that, we focused on how *TDP‐43* contributes to ESCC development.

In line with the TCGA, GSE164158, GSE45670, and the Cancer Cell Line Encyclopedia (CCLE) data (Figure S1, Supporting Information), *TDP‐43* showed increased expression in cancerous tissues in comparation with normal tissues in other ESCC cohorts (GSE38129, GSE77861, and GSE32424) (all *P* < 0.05) (Figure 1C–E). Immunohistochemistry (IHC) data from the Human Protein Atlas (https://www.proteinatlas.org) demonstrated that the increased TDP‐43 protein expression exists in ESCC tissues, but not in normal esophageal tissues (Figure 1F). Furthermore, evidently elevated *TDP‐43* expression in malignant tissues of ESCC patients with advanced diseases (higher cancer grades, higher AJCC stages, or more lymph node metastases) was observed compared to individuals with local diseases (lower cancer grades, lower AJCC stages, or less lymph node metastases) in TCGA (all *P* < 0.001) (Figure 1G–I). Collectively, these findings indicate that *TDP‐43* might function as a novel oncogene in ESCC.

### 3.2 TDP‐43 Promotes ESCC Cell Proliferation In Vitro and In Vivo

**Figure 2:**
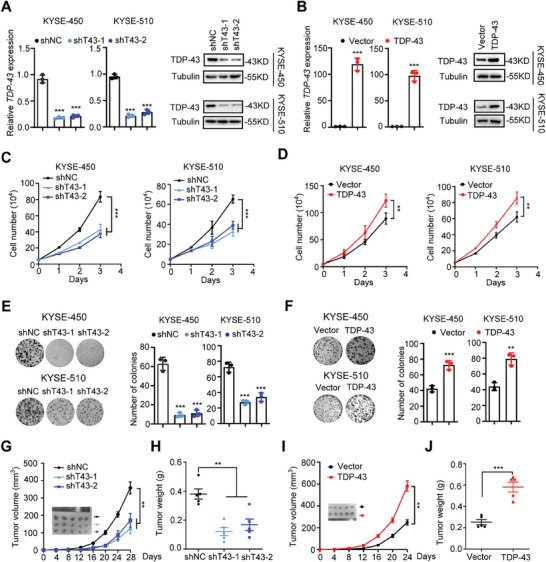
*TDP‐43* promotes proliferation of ESCC cells in vivo and in vitro. A) Relative expression of *TDP‐43* mRNA and protein levels in KYSE‐450 or KYSE‐510 cell lines that stabilized silenced *TDP‐43* (by shRNAs). B) Relative expression of *TDP‐43* mRNA and protein levels in the *TDP‐43*‐OE ESCC cells. C) Silencing of *TDP‐43* inhibited proliferation of KYSE‐450 or KYSE‐510 cells. D) Overexpressed *TDP‐43* promoted proliferation of ESCC cells. E,F) *TDP‐43* promoted clonogenicity of KYSE‐450 or KYSE‐510 cells. G–J) In vivo growth of ESCC xenografts was accelerated by *TDP‐43*. Decreased or increased tumor growth were observed in nude mice via subcutaneous injection of ESCC cells after *TDP‐43*‐knockdown or *TDP‐43*‐overexpression (*n* = 5). Decreased or increased tumor weights were found in nude mice via subcutaneous injection of ESCC cells after *TDP‐43*‐knockdown or *TDP‐43*‐overexpression (*n* = 5). In (A–F) n = 3 biological replicates, G–J) n = 5 biological replicates. Data are the mean ± SD (A–F) and the mean ± SEM (G‐J). The *p‐*value was determined by a two‐tailed unpaired Student's *t*‐test. ^**^ *p* < 0.01, ^***^ *p* < 0.001.

To determine whether *TDP‐43* is functionally involved in ESCC development, we employed lentiviral transduction strategies to either up‐ or down‐regulate *TDP‐43* expression in KYSE‐450 and KYSE‐510 cells (**Figure** **2**A,B; Figure S2A,B, Supporting Information). We found that the *TDP‐43*‐KD KYSE‐450 or KYSE‐510 cells showed inhibited proliferation ability in comparation with controls (all *P* < 0.001) (Figure 2C), whereas the *TDP‐43*‐OE ESCC cells exhibited increased proliferation capability compared to control cells (both *P* < 0.01) (Figure 2D). We also found that silencing of *TDP‐43* significantly promoted apoptosis of ESCC cells (all *P *< 0.01) (Figure S2C,D, Supporting Information). Moreover, we found that depletion of *TDP‐43* obviously increased the expression of the proapoptotic protein BAX and BID, and decreased the expression of antiapoptotic protein BCL2 in ESCC cells (Figure S2E, Supporting Information). That is, knockdown of *TDP‐43* induced apoptosis of ESCC cells and, thus, cell death. We successfully validated oncogenic impacts of *TDP‐43* on KYSE30, KYSE180 and KYSE520 ESCC cell lines (all *P* < 0.01) (Figure S2F–H, Supporting Information). Consistently, clone formation of ESCC cells was suppressed after knocking‐down *TDP‐43* expression (all *P* < 0.001) (Figure 2E); while reinforced clonogenicity of ESCC cells was found after ectopic *TDP‐43* expression (both *P* < 0.01) (Figure 2F). Next, we investigated the effects of *TDP‐43* on ESCC cell motility and invasion. There were no evident impacts of *TDP‐43* on motility and invasion of ESCC cells (Figure S3, Supporting Information). These data elucidate that *TDP‐43* is essential for ESCC cell proliferation, but not for tumor migration and metastasis.

We next assessed the role of *TDP‐43* in vivo using ESCC xenografts through injection of nude mice with the *TDP‐43*‐KD or *TDP‐43*‐OE KYSE‐450 cells. When comparing the *TDP‐43*‐KD xenografts to controls, there were clearly reduced tumor volumes and tumor weights (all *P* < 0.01) (Figure 2G,H). Conversely, the *TDP‐43*‐OE xenografts showed a significant increase in terms of tumor volumes and tumor weights compared to the control group (both *P* < 0.01) (Figure 2I,J). Together, these findings elucidate that *TDP‐43* promotes malignant proliferation of ESCC cells in vitro and in vivo.

### 3.3 TP63 is a Crucial Target Gene of TDP‐43 in ESCC

**Figure 3:**
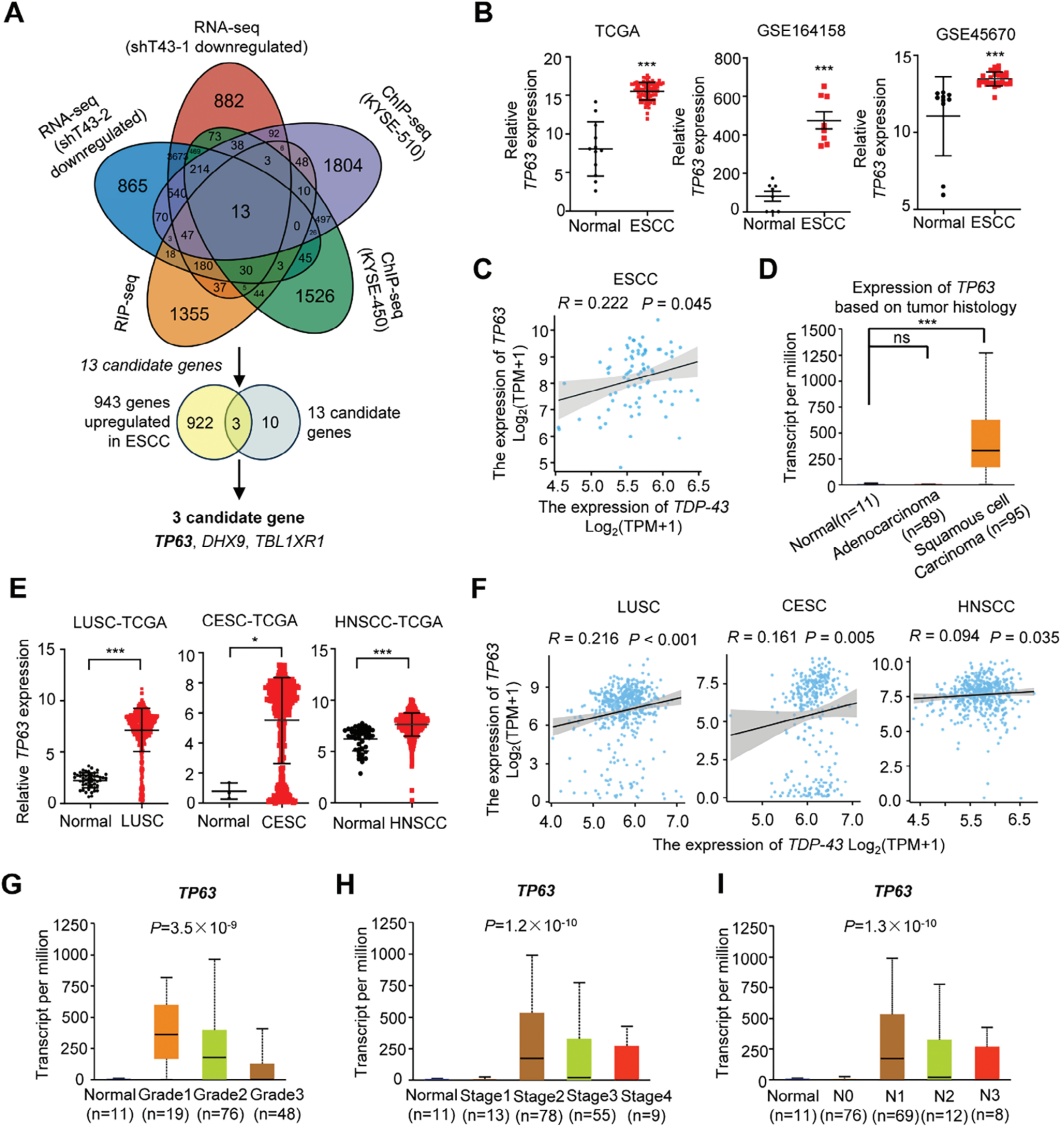
*TP63* is a target gene regulated by TDP‐43. A) A schematic view for identification of the candidate target genes of TDP‐43 in ESCC. B) In the TCGA and GEO cohorts, *TP63* expression levels were higher in ESCC tissues than in normal specimens. C) The expression correlation between *TDP‐43* and *TP63* in ESCC tissues of the TCGA ESCA cohort. D) *TP63* expression levels in different tumor histology of esophageal carcinoma of the TCGA ESCA cohort. E) There were elevated *TP63* expression levels in LUSC, CESC, or HNSCC tissues compared to normal specimens in the TCGA cohorts. F) The expression correlations between *TDP‐43* and *TP63* in LUSC, CESC, or HNSCC tissues of TCGA. G–I) Differential *TP63* expression in cancerous tissues of patients with esophageal carcinoma of different grades (G), clinical stages (H), and lymph nodal metastasis status I) in the TCGA ESCA cohort. Data represent mean ± S.D., the *p‐*value was determined by a two‐tailed paired *t*‐test or unpaired Student's *t*‐test or one‐way ANOVA. The expression correlation between genes were calculated with Pearson's correlation analyses. ns, not significant; ^*^ *P* < 0.05, ^***^ *p* < 0.001.

We then investigate the mechanism that underlies the oncogenic functions of TDP‐43 in ESCC. Considering that TDP‐43 could function as a RBP and/or a TF, we performed RNA‐seq, TDP‐43 RIP‐seq, and TDP‐43 ChIP‐seq in ESCC cells (**Figure** **3**A). For RNA‐seq, the *TDP‐43*‐KD KYSE‐450 cells (shT43‐1 and shT43‐2) and the control cells were utilized. To identify potential TDP‐43‐regulated genes, TDP‐43 RIP‐seq of KYSE‐450 cells as well as TDP‐43 ChIP‐seq in KYSE‐450 and KYSE‐510 cells were also carried out. After integration of the RNA‐seq, RIP‐seq, and ChIP‐seq data, we identified thirteen candidate TDP‐43 target genes in ESCC (Figure 3A). Among these thirteen genes, three genes (*TP63*, *DHX9*, and *TBL1XR1*) showed remarkably higher expression in ESCC tissues than that in normal tissues from the TCGA, GSE164158, or GSE45670 cohorts (all *P* < 0.05) (Figure 3A). To validate the potential regulation of TDP‐43 for these target genes, we measured expression levels of *TP63*, *DHX9*, and *TBL1XR1* in the *TDP‐43*‐KD and wide‐type ESCC cells (Figure S4, Supporting Information). Considering that only *TP63* levels could be evidently suppressed in ESCC cells with silenced *TDP‐43* expression (Figure S4A,B, Supporting Information), we focused on how TDP‐43 regulates *TP63* expression in ESCC.

There was an increase in *TP63* expression in ESCC tissues relative to normal tissues in multiple cohorts (TCGA, GSE164158, or GSE45670, all *P* < 0.001) (Figure 3B). A significantly positive expression correlation between *TDP‐43* and *TP63* expression was observed in ESCC tissues (Figure 3C). Furthermore, *TP63* was highly expressed only in ESCC tissues but not in EAC specimens in contrast to normal tissues (Figure 3D). We also analyzed *TP63* and *TDP‐43* expression levels in three other types of SCCs from TCGA, including lung squamous cell carcinoma (LUSC), cervical squamous cell carcinoma (CESC), and head and neck squamous cell carcinoma (HNSCC). Interestingly, there were higher levels of *TDP‐43* and *TP63* expression in tissues of LUSC, CESC, or HNSCC than those in normal tissues (all *P* < 0.05) (Figure 3E; Figure S5, Supporting Information). Moreover, positive correlations between *TDP‐43* and *TP63* expression were observed in TCGA LUSC, CESC or HNSCC tissues (all *P*  <  0.05) (Figure 3F). Notably, there were strikingly up‐regulated *TP63* levels in cancerous specimens of ESCC patients with advanced diseases (higher cancer grades, higher AJCC stages, or more lymph node metastases) compared with those of subjects with local diseases (lower cancer grades, lower AJCC stages, or less lymph node metastases) in TCGA (all *P *< 0.001) (Figure 3G–I). These data indicate that oncogenic *TP63* might be a key target gene of TDP‐43 protein in ESCC cells.

### 3.4 TF TDP‐43 Promotes TP63 Transcription in ESCC

**Figure 4:**
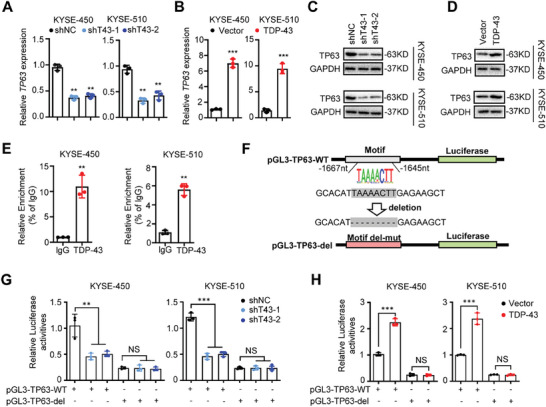
TDP‐43 transcriptionally up‐regulates *TP63* expression. A) Relative expression of *TP63* mRNAs after knock‐down of *TDP‐43* by shRNAs in KYSE‐450 and KYSE‐510 cells. B) Relative *TP63* mRNA expression in the *TDP‐43*‐OE ESCC cells. C,D) Western blot assays showed TP63 protein levels in KYSE‐450 and KYSE‐510 cells that stabilized either silenced *TDP‐43* or overexpressed *TDP‐43*. E) The ChIP assays showed that there was evident binding of TDP‐43 to the *TP63* promoter in ESCC cells. F–H) The dual luciferase reporter assays indicated that the TDP‐43‐binding motif is essential for the *TP63* promoter activities in KYSE‐450 and KYSE‐510 cells. Data represent mean ± S.D., the *p‐*value was determined by a two‐tailed unpaired Student's *t*‐test. ns, not significant; ^**^ *p* < 0.01, ^***^ *p* < 0.001. Data shows one representative of three independent experiments with three biological replicates.

To examine the mechanism of how TDP‐43 transcriptionally up‐regulates *TP63* expression, we first detected *TP63* mRNA levels in either *TDP‐43*‐KD or *TDP‐43*‐OE ESCC cell lines (**Figure** **4**A,B). Interestingly, silenced *TDP‐43* evidently reduced *TP63* mRNA levels in KYSE‐450 and KYSE‐510 cells (all *P* < 0.01) (Figure 4A). Conversely, enforced *TDP‐43* expression increased *TP63* mRNA levels in ESCC cells (both *P*<0.001) (Figure 4B). We then measured TP63 protein levels in either *TDP‐43*‐KD or *TDP‐43*‐OE ESCC cells (Figure 4C,D). Consistently, TDP‐43 led to significantly elevated TP63 protein levels in ESCC cells (all *P*<0.001) (Figure 4C,D; Figure S6A,B, Supporting Information).

ChIP‐qPCR analyses showed evident TF TDP‐43 enrichments in the *TP63* promoter of KYSE‐450 and KYSE0510 cell lines (both *P* < 0.01) (Figure 4E). We further investigated how TF TDP‐43 impacts *TP63* promoter activities using reporter gene assays (Figure 4F). It has been shown that the promoter activities of the pGL3‐TP63‐WT construct with the *TP63* promoter containing the TDP‐43 binding motif were significantly higher than those of the pGL3‐TP63‐del construct without the TF TDP‐43 binding motif in ESCC cells (Figure 4G–H). In particular, an obvious decrease of pGL3‐TP63‐WT luciferase activities in the *TDP‐43*‐KD cells was found compared to the control cells (all *P *< 0.01) (Figure 4G). Nevertheless, the *TDP‐43*‐KD cells transfected with pGL3‐TP63‐del did not show a similar decline in luciferase activities (Figure 4G). By contrast, enforced *TDP‐43* expression markedly elevated luciferase activities of the pGL3‐TP63‐WT construct in ESCC cells (both *P *< 0.001). However, there was no such increased luciferase activities in the *TDP‐43*‐OE cells transfected with pGL3‐TP63‐del (Figure 4H). The rescue assays showed that overexpression of *TDP‐43* in the ESCC cells with silenced *TDP‐43* markedly restored luciferase activities of the pGL3‐TP63‐WT construct (Figure S6C,D, Supporting Information). Taken together, these results indicated that TF TDP‐43 binds to the *TP63* promoter and activates *TP63* expression in ESCC cells.

### 3.5 RBP TDP‐43 Stabilizes TP63 mRNAs in ESCC

**Figure 5:**
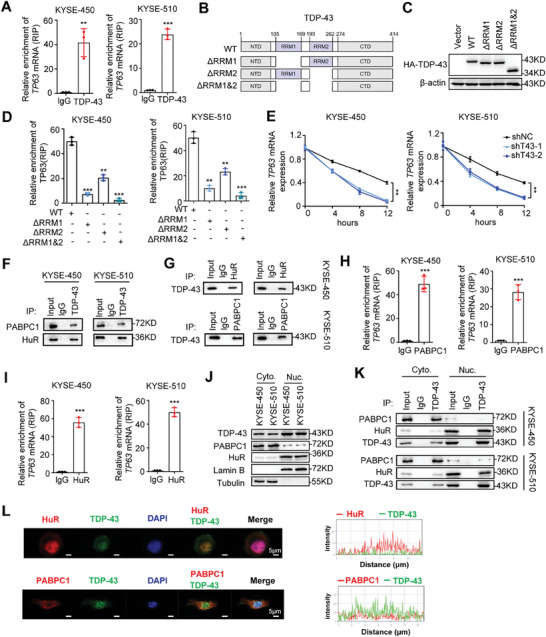
RBP TDP‐43 stabilizes *TP63* mRNAs via recruiting PABPC1 in cytoplasm or HuR in nucleus. A) The RIP assays showed relative enrichments of *TP63* mRNAs associated with endogenous TDP‐43 relative to an input control in KYSE‐450 or KYSE‐510 cells. IgG served as the control. B,C) RBP TDP‐43 protein domains and various truncated *TDP‐43* expression plasmids. Western blot assays showed expression of full length or truncated TDP‐43 proteins after cells transfected HA‐tagged plasmids encoding full length TDP‐43 or truncated TDP‐43. D) The RIP assays were performed using KYSE‐450 or KYSE‐510 cells transfected HA‐tagged plasmids encoding full length TDP‐43 or truncated TDP‐43. Relative enrichments of *TP63* mRNAs associated with different HA‐tag‐TDP‐43 relative to an input control were shown. IgG served as the control. E) The *TDP‐43*‐KD KYSE‐450 or KYSE‐510 cells and the control cells were treated with actinomycin D. The *TP63* mRNAs levels were determined by qRT‐PCR at different time points. F,G) The Co‐IP assays showed interactions between TDP‐43 and PABPC1 or TDP‐43 and HuR in KYSE‐450 and KYSE‐510 cells. H,I) The RIP‐qPCR analysis showed relative enrichments of *TP63* mRNAs associated with endogenous PABPC1 or HuR relative to an input control in ESCC cells. IgG served as the control. J) Western blot assays showed expression of PABPC1 or TDP‐43 and HuR in in cytoplasm and the nucleus of KYSE‐450 or KYSE‐510 cells. K) The Co‐IP assays showed that TDP‐43 binds to HuR in the nucleus and binds to PABPC1 in the cytoplasm of KYSE‐450 or KYSE‐510 cells. L) The immunofluorescence assays showed co‐localization of TDP‐43 and PABPC1 or TDP‐43 and HuR proteins in ESCC cells. Data represent mean ± S.D., the *p‐*value was determined by a two‐tailed unpaired Student's *t*‐test. ns, not significant; ^**^ *p* < 0.01, ^***^ *p* < 0.001. Data shows one representative of three independent experiments with three biological replicates.

Besides acting as a TF, TDP‐43 is also a multifunctional RBP controlling RNA metabolism. To further investigate whether TDP‐43 is involved in stabilizing *TP63* mRNAs as an RBP, we performed RIP‐qPCR to verify the binding of TDP‐43 with *TP63* mRNAs in ESCC cells. In KYSE‐450 and KYSE‐510 cells, there were significant enrichments of *TP63* mRNAs in RNPs precipitated with antibody against TDP‐43 as compared with the IgG control (**Figure** **5**A). RBP TDP‐43 contains two RNA recognition motif domains (RRM1 and RRM2), a nuclear localization signal (NLS) at N‐terminal domain (NTD), as well as two intrinsically disordered regions (IDR1 and IDR2) at the C‐terminal domain (CTD) (Figure 5B).^[^ ^31^ ^]^ To explore the specific domains required for the interaction between TDP‐43 protein and *TP63* mRNAs, we constructed various truncated *TDP‐43* expression plasmids (Figure 5B,C). RIP‐qPCR assays revealed that the RRM‐1 domain (aa105‐169) and the RRM2 domain (aa193‐262) are required for binding of RBP TDP‐43 to *TP63* mRNAs (Figure 5D). Next, we examined whether TDP‐43 affects the RNA stability of *TP63* mRNAs in ESCC cells. Following actinomycin D treatments, the half‐life of *TP63* mRNAs was markedly shortened in *TDP‐43*‐KD KYSE‐450 and KYSE‐510 cells in comparison with the control cells (both *P*<0.01) (Figure 5E).

Accumulated evidences indicated that multiple RBPs play essential part in maintaining RNA stability. Therefore, we hypothesized that TDP‐43 may affect *TP63* mRNAs stability through recruitment of other RBPs to target RNAs. Using Co‐IP plus mass spectrometry (Co‐IP/MS), we identified PABPC1 and HuR, two well‐known RBPs involved in stabilizing mRNAs (Table S4, Supporting Information). Co‐IP assays consistently demonstrated that endogenous TDP‐43 in KYSE‐450 and KYSE‐510 cells could be precipitated with PABPC1 or HuR (Figure 5F). Additionally, endogenous PABPC1 or HuR could also be precipitated with TDP‐43 in ESCC cells (Figure 5G). RIP‐qPCR assays revealed significant enrichments of *TP63* mRNAs in RNPs precipitated with antibody against PABPC1 or HuR in cells (Figure 5H,I). We then measured PABPC1 and HuR levels in cytoplasm and the nucleus of ESCC cells after separating the cytoplasm and the nucleus proteins of cells (Figure 5J). Interestingly, PABPC1 is mainly in the cytoplasm of cells, but HuR is dominantly in nucleus of cells (Figure 5J). Co‐IP assays showed that TDP‐43 interacts with HuR in the nucleus and interacts with PABPC1 in the cytoplasm (Figure 5K). In line with this, PABPC1 and TDP‐43 co‐localized mainly in the cytoplasm of KYSE450 and KYSE510 cells, in accordance with immunofluorescence assays (Figure 5L). However, HuR and TDP‐43 co‐localized mainly in nucleus of ESCC cells (Figure 5L). To investigate whether PABPC1 and HuR proteins are necessary for TDP‐43 to regulate *TP63* mRNA stability, we examined half‐life of *TP63* mRNAs in ESCC cells treated with actinomycin D (Figure S7, Supporting Information). The half‐life of *TP63* mRNAs was significantly shortened in *TDP‐43*‐KD ESCC cells with silenced *PABPC1* or *HuR* compared to the control cells (Figure S7, Supporting Information). Collectively, these data suggest that TDP‐43 recruits PABPC1 in the cytoplasm or HuR in the nucleus to *TP63* mRNAs, facilitates *TP63* mRNA stability in cells, and, thus, post‐transcriptionally elevates TP63 expression.

### 3.6 The Positive Feedback Regulation of TF TP63 on TDP‐43 Transcription

**Figure 6:**
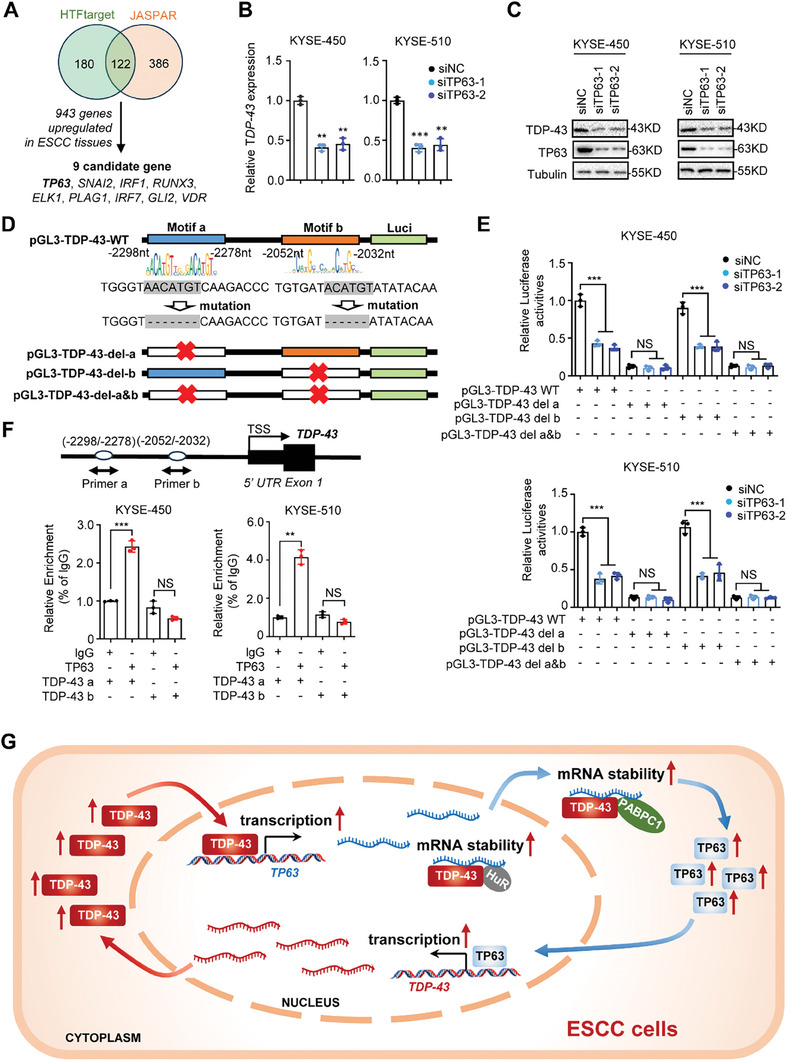
The master TF TP63 promotes transcription and expression of *TDP‐43*. A) Venn diagram analyses indicated potential TFs involved in transcriptional regulation of *TDP‐43* by HTFtarget and JASPAR. B) Relative expression of *TDP‐43* mRNAs after knock‐down of *TP63* by siRNAs in KYSE‐450 and KYSE‐510 cells. C) Western blot assays showed expression of TDP‐43 protein after knock‐down of *TP63* by siRNAs in ESCC cells. D,E) The dual luciferase reporter assays indicated that the TP63‐binding Motif a is essential for the *TDP‐43* promoter activities. F) The ChIP‐qPCR analyses indicated evident enrichments of TF TP63 in the Motif a of the *TDP‐43* promoter in ESCC cells. G) Graphical representation of the regulation and functions of TDP‐43 and TP63 in ESCC. TDP‐43 facilitated *TP63* transcription and mRNA stability, which in turn markedly enhanced *TP63* expression. The master TF TP63 in turn transcriptionally activated the *TDP‐43* expression. The TDP‐43/TP63 positive feedback circuit promotes ESCC progression. Data represent mean ± S.D., the *p‐*value was determined by a two‐tailed unpaired Student's *t*‐test. ns, not significant; ^**^ *p* < 0.01, ^***^ *p* < 0.001. Data shows one representative of three independent experiments with three biological replicates.

In light of the importance of TDP‐43 in ESCC, we investigated how *TDP‐43* expression is regulated in cells. Using JASPAR (https://jaspar.genereg.net/) and hTFtarget (http://bioinfo.life.hust.edu.cn/hTFtarget#!/) algorithms, we firstly predicted the potential TFs binding to the 2500 bp *TDP‐43* promoter region (−1 to −2500 bp from TSS) (**Figure** **6**A). The expression levels of the one hundred and twenty‐two candidate TF genes were then profiled in ESCC tissues from the TCGA ESCC cohort and two ESCC cohorts (GSE164158 and GSE45670). Nine candidate TFs, including TP63, with significantly high expression in cancerous tissues were then identified (Figure 6A). It is worth noting that TP63 acts as a master TF during ESCC development. Therefore, we examined whether TF TP63 transcriptionally impacts *TDP‐43* expression in ESCC cells. In fact, in KYSE‐450 and KYSE‐510 cells, silencing *TP63* led to markedly decreased levels of *TDP‐43* mRNA and protein (Figure 6B,C; Figure S8A, Supporting Information).

We next investigated whether *TDP‐43* is a transcriptional target gene of TP63 in ESCC. There are two putative TF TP63 binding motifs (Motif a and Motif b) in the *TDP‐43* promoter. To evaluate whether these motifs confer TP63‐dependent transcriptional activation of *TDP‐43*, we cloned the *TDP‐43* promoter containing the wild‐type motifs into pGL3‐basic (pGL3‐TDP‐43‐WT). Three mutant constructs (pGL3‐TDP‐43‐del‐a, pGL3‐TDP‐43‐del‐b, and pGL3‐TDP‐43‐del‐a&b) were obtained after deletion of Motif a, Motif b, or both motifs from pGL3‐TDP‐43‐WT (Figure 6D). Dual luciferase reporter gene assays elucidated that silencing of *TP63* expression led to reduced *TDP‐43* promoter activities of KYSE‐450 and KYSE‐510 cells transfected with pGL3‐TDP‐43‐WT (both *P* < 0.001) (Figure 6E). Similar results were observed in cells transfected with pGL3‐TDP‐43‐del‐b (both *P* < 0.001) (Figure 6E). However, no such decrease in luciferase activities was observed in ESCC cells transfected with pGL3‐TDP‐43‐del‐a or pGL3‐TDP‐43‐del‐a&b (all *P* > 0.05) (Figure 6E). These findings revealed that the TP63 binding Motif a is essential for the *TDP‐43* promoter. To further validate this, ChIP‐qPCR assays were performed using antibodies against TP63 or IgG. As shown in Figure 6F, evident enrichments of TF TP63 in the Motif a of the *TDP‐43* promoter were observed, whereas no such enrichments existed at Motif b. The rescue assays indicated that the crosstalk between two TFs, TDP‐43, and TP‐63, are necessary for ESCC cell proliferation (Figure S8B–E, Supporting Information). Taken together, these data demonstrated that TF TP63 transcriptionally activates *TDP‐43* expression through its binding of the Motif a in ESCC.

## 4 Discussion

RBPs are a class of highly conserved proteins that regulates RNA stability, RNA splicing, RNA alternative polyadenylation, and RNA transport during tumorigenesis.^[^ ^7^, ^32^, ^33^, ^34^ ^]^ In the current study, we found that RBP TDP‐43 was highly expressed in ESCC and associated with poor prognosis of patients. In ESCC cells, the master TF TP63 of SCCs induced high levels of *TDP‐43* transcription and elevated *TDP‐43* expression. TDP‐43 promoted proliferation of ESCC cells in vitro and in vivo. Mechanistically, TDP‐43 interacted with PABPC1 and HuR proteins in nucleus, recruited them to *TP63* mRNAs, facilitated *TP63* mRNA stability, and elevated TP63 expression at post‐transcriptional level. More importantly, we found that TDP‐43 also acted as a crucial TF binding to the *TP63* promoter and transcriptionally activated *TP63* expression in ESCC cells. Notably, there was a significantly positive correlations between *TDP‐43* and *TP63* expression in ESCC tissues from multiple patient cohorts. These findings indicate that the TDP‐43/TP63 positive feedback circuit plays a part in ESCC progression (Figure 6G).

Multiple RBPs are involved in gastrointestinal cancer progression.^[^ ^35^, ^36^, ^37^ ^]^ For instance, we found that DDX5, as a multifunctional RBP, can interact with lncPSCA to regulate the transcription of the P53 signaling genes, thereby promoting gastric cancer cell growth and metastasis.^[^ ^28^ ^]^ It has been reported that TDP‐43 functions as a DNA/RNA‐binding protein.^[^ ^14^ ^]^ In our previous study, we found that TDP‐43, which was markedly up‐regulated in NSCLC tissues, interacted with lncRNA LCETRL3 to cause NSCLC cells to be resistant to EGFR‐TKIs.^[^ ^38^ ^]^ LncRNA LCETRL3 could inhibit TDP‐43 protein ubiquitination, avoid TDP‐43 proteasome‐degradation, and increase NOTCH1 levels, resulting in activation of the downstream AKT signaling.^[^ ^38^ ^]^ However, it is still unclear how TDP‐43 contributes to ESCC development. Indeed, we disclosed that *TDP‐43* acts as a novel oncogene via boosting the master TF *TP63* expression transcriptionally and post‐transcriptionally in ESCC, which developed the knowledge about the importance of TDP‐43‐controlled *TP63* expression.

As a TF of the P53 family, TP63 plays an oncogenic role in SCCs.^[^ ^17^, ^39^, ^40^, ^41^ ^]^ Consistently, markedly elevated levels of *TP63* were observed in LUSC, CESC, and HNSCC specimens compared to normal tissues according to TCGA. For ESCC, the genomic analyses revealed that *TP63* is frequently amplified in cancerous tissues and is significantly up‐regulated in comparison with non‐tumor and adenocarcinoma tissues.^[^ ^42^ ^]^ For example, TP63 can bind to promoters of *DKK3*, *LINC01503* and/or *RSK4* to drive high expression of these oncogenes in ESCC.^[^ ^23^, ^43^, ^44^ ^]^ In line with these, we found that TP63 could transcriptionally enhance *TDP‐43* expression and, thus, promote ESCC progression, which broadens the understanding of mechanisms by which the master TF TP63 functions.

PABPC1 and HuR are two highly conserved RBPs in eukaryotes and have elevated expression in multiple cancers.^[^ ^33^, ^45^, ^46^ ^]^ PABPC1 is a member of the poly(A)‐binding proteins (PABPs) protein family, which binds to the poly(A) tail of eukaryotic mRNAs. PABPC1, a highly conserved RBP in species ranging from yeast to human, locates dominantly in cytoplasm, coats the 3′ end of cytoplasmic mRNAs with very high affinity, packages the mRNAs into poly(A) RNPs, physically protects the transcripts from unspecific degradation.^[^ ^47^, ^48^ ^]^ It has been reported that PABPC1 could promote ESCC progression through stabilizing *IFI27* mRNAs.^[^ ^49^ ^]^ Importantly, we found that TDP‐43 and PABPC1 interacted in ESCC cells, which improved PABPC1 binding to *TP63* mRNAs, increased *TP63* expression, and contributed to esophageal tumorigenesis. The idea that TDP‐43 interactions with PABPC1 in cytoplasm helps to explain why TDP‐43 might favor stabilization of mRNAs in ESCC. HuR, also known as ELAVL1, binds transcripts with adenyl‐uridine‐rich elements (AREs) within the 3′‐UTRs or introns.^[^ ^50^ ^]^ HuR is predominantly found in the nucleus and plays an essential role in maintaining RNA stability, such as *P53*,^[^ ^46^ ^]^ *VEGF*,^[^ ^51^ ^]^ and *NEAT1*.^[^ ^52^ ^]^ Consistently, we identified multiple AREs within the *TP63* mRNA 3′‐UTR (Figure S9, Supporting Information) and found evident enrichments of *TP63* mRNAs‐precipitated by HuR in ESCC. Our findings revealed a novel TDP‐43‐mediated architecture that can protect transcripts from degradation via binding and recruiting different RBPs in cytoplasm or nucleus, with *TP63* as an example.

In conclusion, we found that *TDP‐43* is a novel oncogene in ESCC, showing markedly elevated expression in malignant tissues and associated with poor prognosis of patients. On one hand, TDP‐43 functions as an RBP by reducing degradation of *TP6*3 mRNAs and as a TF by prompting *TP63* transcription. Notably, TDP‐43 binds PABPC1 in cytoplasm or HuR in nucleus to stabilize *TP63* mRNAs, though no such role of RBPs has been reported during tumorigenesis. On the other hand, the master TF TP63 of SCCs facilitates *TDP‐43* transcription and expression, which creates a TDP‐43/TP63 positive feedback circuit in ESCC. Our findings extend the present understanding of RBPs and imply the clinical potential of TDP‐43 as novel therapeutic targets for ESCC.

## Conflict of Interest

The authors declare no conflict of interest.

## Author Contributions

W.L. and Y.Y. contributed equally to this work. M.Y. conceived, designed, and supervised this study. W.L., Y.Y., and M.Y. acquired, analyzed, and interpreted the data from experiments. L.H., X.Y., T.W., and N.Z. provided technique supports. W.L., Y.Y., and M.Y. were engaged in statistical and bioinformatics analyses. M.Y., Y.Y., and N.Z. drafted the manuscript. M.Y. and N.Z. critically revised the manuscript for important intellectual content.

## Supporting information

Supporting Information

## Data Availability

The data that support the findings of this study are available from the corresponding author upon reasonable request.
