# Supplementary material for: The TDP‐43/TP63 Positive Feedback Circuit Promotes Esophageal Squamous Cell Carcinoma Progression
Source: Adv Sci (Weinh). 2024 Jul 18;11(35):2402913. doi: 10.1002/advs.202402913 (PMC11425248; doi:10.1002/advs.202402913)
Supplement: Supplementary file 1 — Supporting Information [file ADVS-11-2402913-s001.pdf]

Figure S1

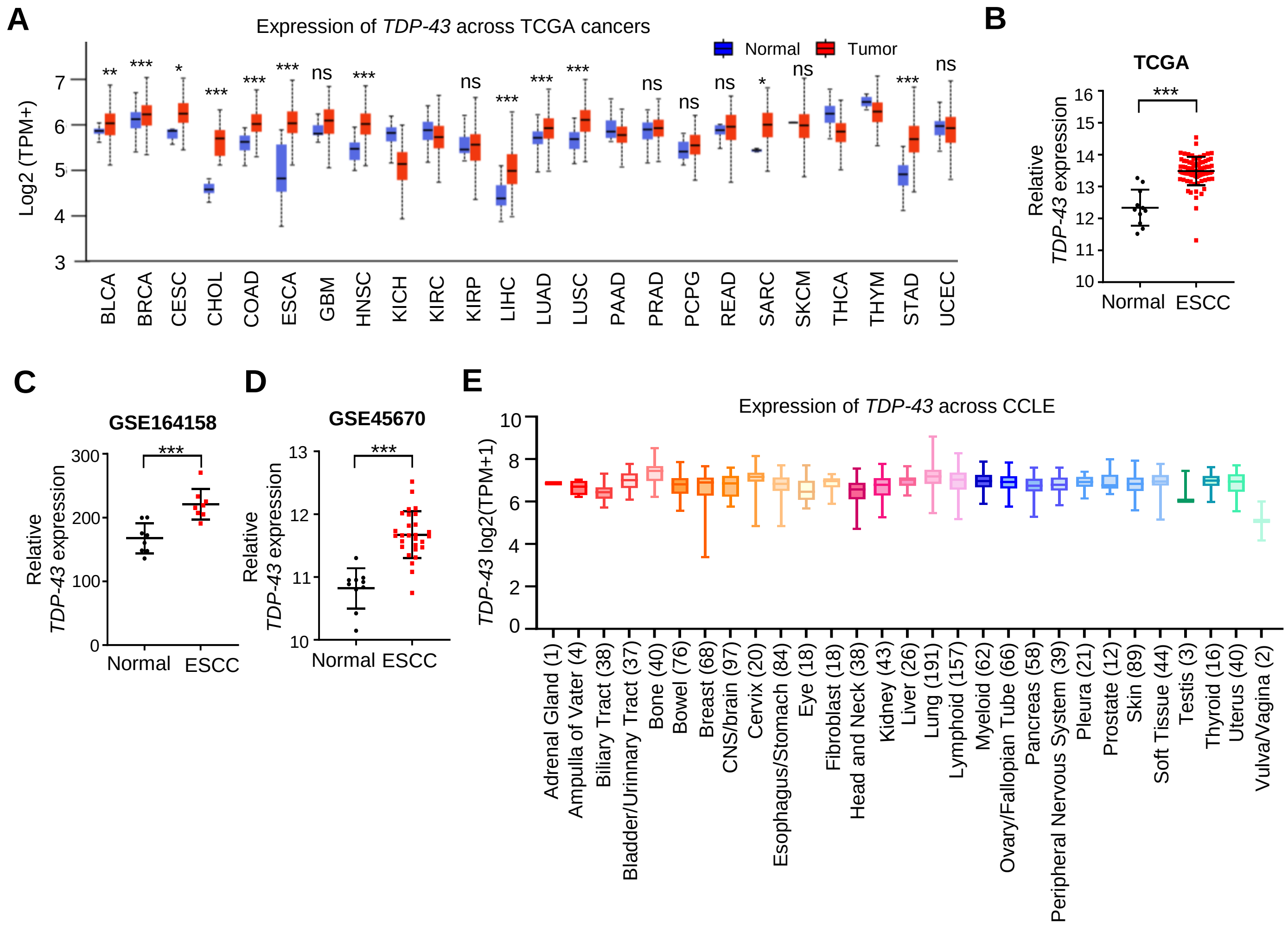

Figure S1. TDP-43 is highly expressed in many cancers. A, Expression of TDP-43 were evaluated in multiple tumor tissue types from the TCGA database. B-D, Expression of TDP-43 was higher in ESCC tissues than in adjacent normal tissues (TCGA, GSE164158, and GSE45670). E, TDP-43 expression levels were evaluated in multiple cancer cell lines from the CCLE database.

Figure S2

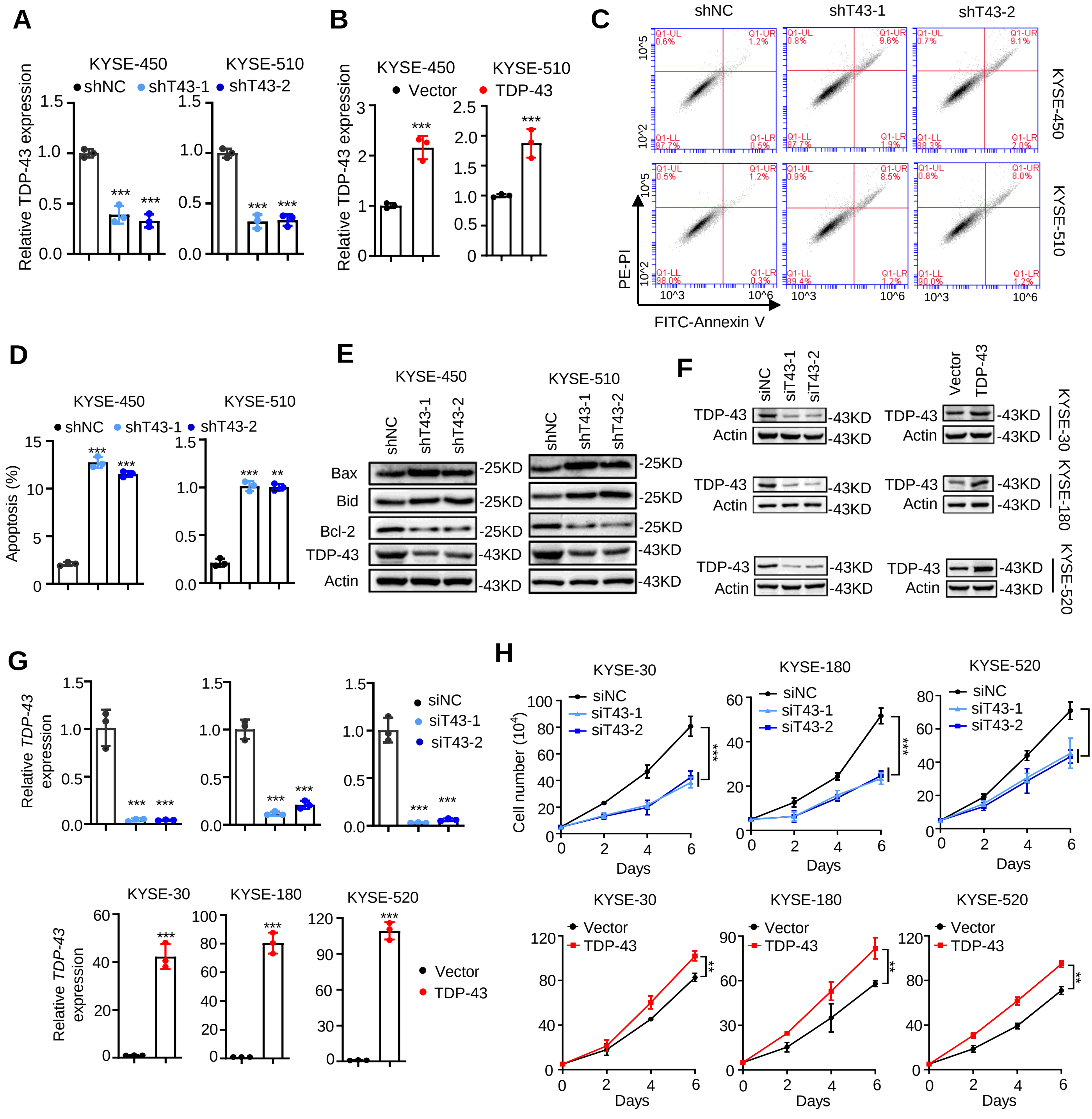

A
KYSE-450
KYSE-510
shNC
shT43-1
shT43-2
Relative TDP-43 expression
B
Vector
TDP-43
C
shNC
shT43-1
shT43-2
KYSE-450
KYSE-510
PE-PI
FITC-Annexin V
D
Apoptosis (%)
E
Bax
Bid
Bcl-2
TDP-43
Actin
-25KD
-43KD
F
siNC
siT43-1
siT43-2
KYSE-30
KYSE-180
KYSE-520
G
Relative TDP-43 expression
H
Cell number (10^4)
Days

Figure S2

Figure S2. TDP-43 promotes proliferation of ESCC cells. A, TDP-43 was significantly knockdown using shT43-1 or shT43-2 in KYSE-450 and KYSE-510 cells. B, TDP-43 was significantly overexpressed in KYSE-450 and KYSE-510 cells. C-D, Flow cytometry assays indicated that silencing of TDP-43 increased ESCC cell apoptosis. E, Depletion of TDP-43 significantly increased the expression of the proapoptotic protein BAX and Bid, and decreased the expression of antiapoptotic protein BCL2. F, Relative expression of TDP-43 protein levels in KYSE-30, KYSE-180 or KYSE-520 cell lines that transfected with TDP-43 siRNAs. G, Relative expression of TDP-43 mRNA levels in the TDP-43-OE and TDP-43-KD ESCC cells. H, Silencing of TDP-43 inhibited proliferation of KYSE-30, KYSE-180 or KYSE-520 cells; whereas overexpressed TDP-43 promoted proliferation of ESCC cells.

Figure S3

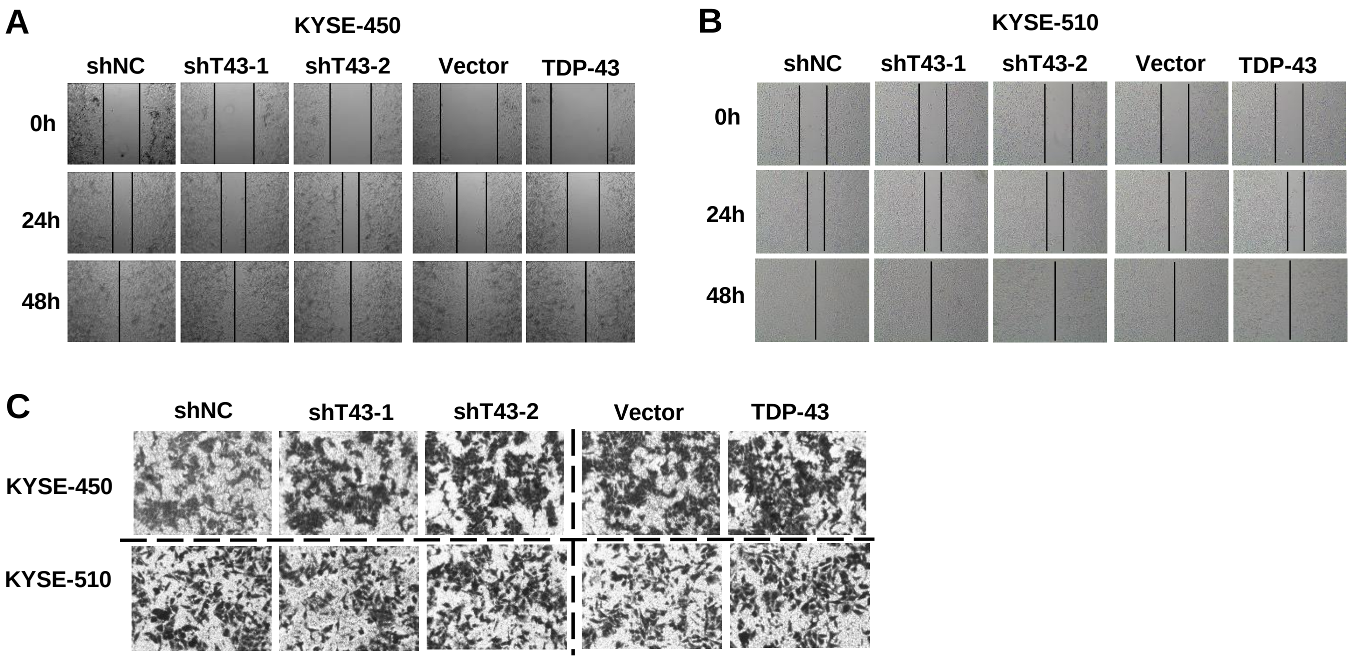

Figure S3. TDP-43 had no effects on ESCC cell migration and invasion. A-B, In KYSE-450 and KYSE-510 cells, *TDP-43*-knockout or the stably enforced *TDP-43* expression have no impacts on wound-healing. The dashed lines indicate the edges of the cell layers. C, TDP-43 did not impact the invasion ability of ESCC cells. Cells on the lower surface of the chamber were stained by crystal violet.

Figure S4

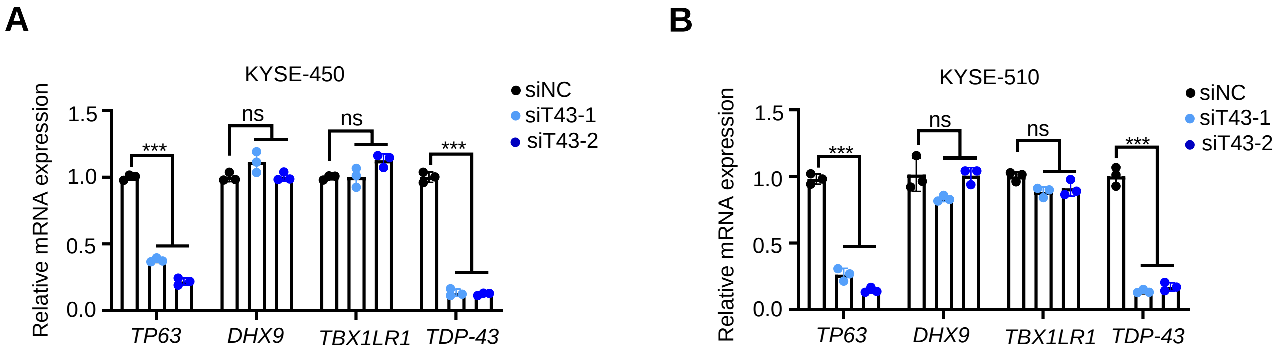

Figure S4. Identification of target genes of TDP-43 in ESCC cells. Knock-down of *TDP-43* markedly down-regulated *TP63* expression, but did not impact *DHX9* and *TBL1XR1* expression in KYSE-450 (A) and KYSE-510 cells (B).

Figure S5

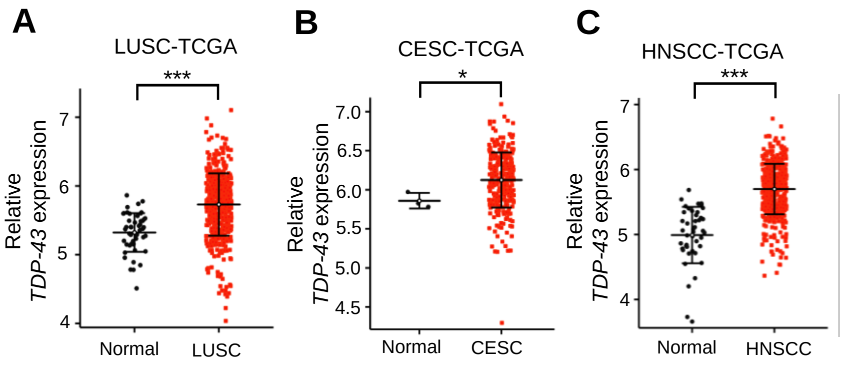

Figure S5. The expression of *TDP-43* in TCGA LUSC, CESC, and HNSCC datasets. A, Relative expression of *TDP-43* in paired LUSC specimens and normal tissues from TCGA. B, Relative expression of *TDP-43* in paired CESC specimens and normal tissues from TCGA. C, Relative expression of *TDP-43* in paired HNSCC specimens and normal tissues from TCGA.

Figure S6

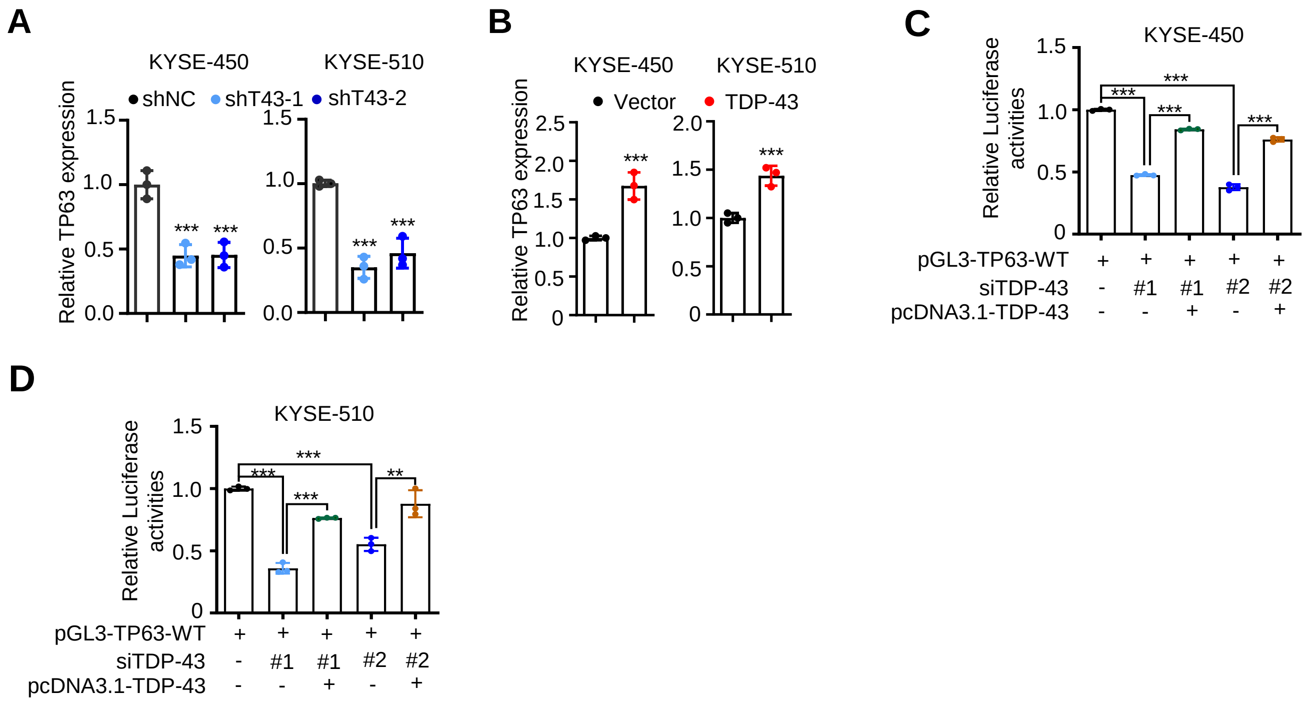

Figure S6. TF TDP-43 transcriptionally up-regulates TP63 expression. A, Knock-down of *TDP-43* markedly down-regulated TP63 expression in KYSE-450 and KYSE-510 cells. B, Overexpression of *TDP-43* significantly up-regulated TP63 expression in ESCC cells. C-D, The dual luciferase reporter assays indicated that the TDP-43-binding motif is essential for the *TP63* promoter activities in ESCC cells.

Figure S7

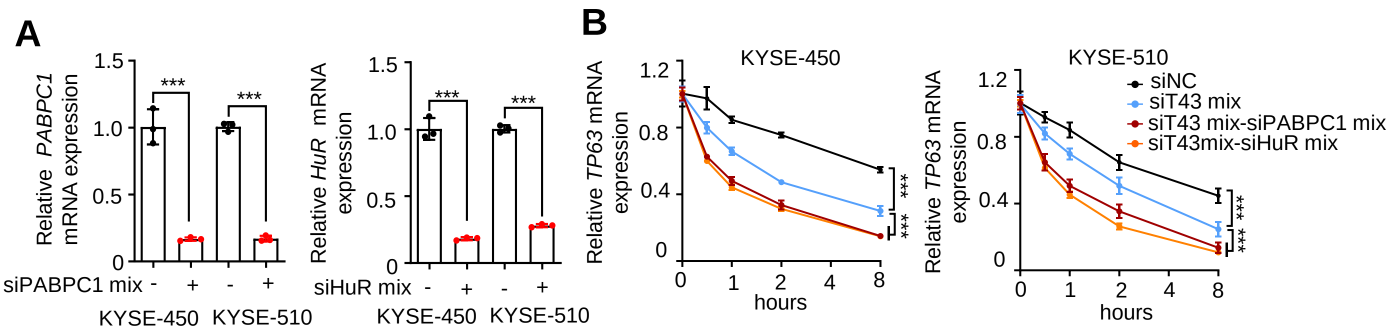

Figure S7. PABPC1 and HuR proteins are necessary for TDP-43 to regulate *TP63* mRNA stability in ESCC cells. A, PABPC1 and HuR was significantly knockdown using siRNA mix in KYSE-450 and KYSE-510 cells. B, The *TDP-43*-KD KYSE-450 or KYSE-510 cells transfected with PABPC1 or HuR siRNA mix were treated with actinomycin D. The *TP63* mRNAs levels were determined by qRT-PCR at different time points.

Figure S8

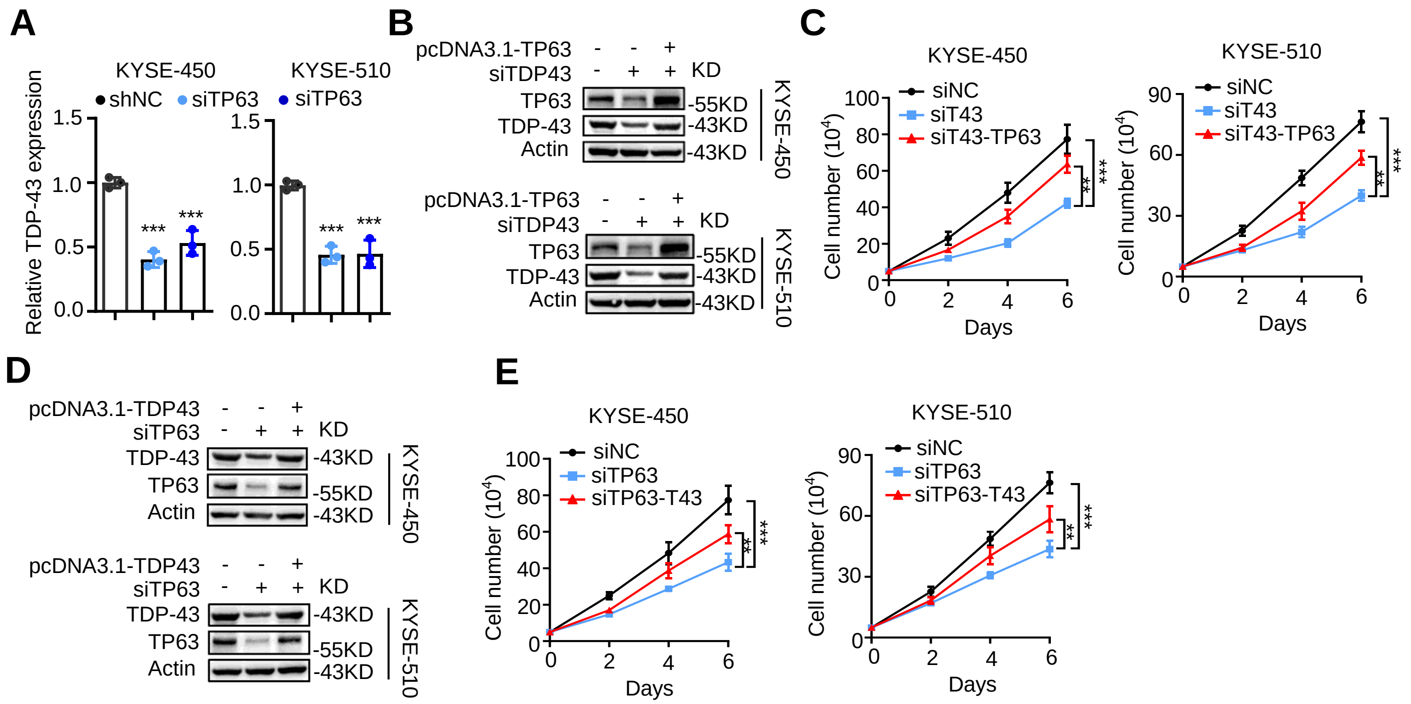

Figure S8. The TDP-43/TP63 positive feedback circuit in ESCC. A, Silencing of *TP63* suppresses TDP-43 expression in ESCC cells. B-C, Depletion of *TDP-43* reduced TP63 expression and inhibited cell proliferation, while *TP63* overexpression restored TDP-43 expression and promoted ESCC cell growth. D-E, Loss of *TP63* reduced TDP-43 expression and inhibited cell proliferation, while overexpression of *TDP-43* restored TP63 expression and promoted ESCC cell growth.

Figure S9

gccucaccaugugagcucuuccuaucccucuccuaacugccagcccccuaaaagcacuccugcuuaaucuucaaagccuucucccuagcuccucccccuuccucuu
gucugauuucuuaggggaaggagaaguaagaggcuaccucuuaccuaacaucugaccuggcaucuaauucugauucuggcuuuaagccuucaaaacuauagcu
ugcagaacuguagcugccauggcuagguagaagugagcaaaaaagaguuggguguccuuaagcugcagagauuucucauugacuuuuauaaagcauguuca
cccuuauagucuaagacuauauauauaaauguauaaauauacaguauagauuuuuggguggggggcauugaguauuguuuaaaaugua auuua aaugaaaga
aaauugaguugcacuuauugaccauuuuuua auuua cuuguuuuggauggcuugucuauacuccuucccuuaaggguaucauguauggugauagguaucua
gagcuuaaugcuacaugugagugacgaugauguacagauucuuucaguucuuuggauucuaaauacaugccacaucaaaccuuugaguagauccauuuccauu
gcuuauuauguagguaagacuguagauauguauucuuuucucaguguugguau auuuua uauuacugacauuucuucuagugaugaugguucacguuggggu
g auuua auccaguuauaagaagaaguucauguccaaacguccucuuuaguuuuugguugggaaugaggaaaauucuuaaaaggcccauagcagccaguucaaa
aacacccgacgucauguauuugagcauaucaguaacccccuuaa auuua auaccagauaccuuaucuuacaauauugauugggaaacauuugcugccauuac
agagguauuaaaacuaaauuucacuacuagauugacuaacucaaauacacauuugcuacuguuguaagaauucugauugauuugauugggaugaaugccauc
uaucuaguucuaacagugaaguuuuacugucuauuaauauucaggguaaauaggaaucauucagaaauguugagucuguacuaaacaguaagauaucucaau
gaaccauaaauucaacuuuguaaaaaucuuuugaagcauagauaauauuguuugguaaauguuucuuuuguuugguaaauguuucuuuuaaagacccuccua
uucuauaaaacucugcauguagaggcuuguuuaccuuucucucucuaagguuuacaauaggaguggugauuugaaaauauaaaauuaugagauugguuuuc
cuguggcauaaauugcaucacuguaucauuuucuuuuuuaaccgguaagaguuucaguuuguuggaaaguaacugugagaacccaguucccguccaucuccc
uuagggacuacccauagacaugaaaggucccacagagcaagagauaagucuuucauggcugcuguugcuuaaaccacuuaaacgaagaguucccuugaaacu
uugggaaaacauguuaaugacaauauuccagaucuuucagaaauauaacacauuuuuuugcaugcaugcaaaugagcucugaaaucuucccaugcauucuggu
caagggcugucauugcacauaagcuuccauuuuaauuuuaaagugcaaaagggccagcguggcucuaaaagguaauguguggauugccucugaaaagugugua
uauauuuuguguggaaauugcauacuuuguauuuugauuauuuuuuuuucuucuugggauagugggauuuccagaaccacacuugaaaccuuuuuuuaucgu
uuuuguauuuucaugaaaauaccauuuaguaagaauaccacaucaaauaagaaauaaugcuaca auuuua agaggggagggaagggaaaguuuuuuuuuauu
auuuuuuuaaaauuuuguauguuaaagagaaugaguccuugauuucaaaguuuuguuguacuuaaaugguaauaagcacuguaaacuucugcaacaagcaug
cagcuuugcaaacccauuaaggggaagaaugaaagcuguuccuugguccuaguaagaagacaaacugcuucccuuacuuugcugaggguuugaauaaaccuag
gacuuccgagcuaugucaguacuauucagguaacacuagggccuuggaaauuccuguacugugucucauggauuuggcacuagccaaagcgaggcacccuuac
uggcuuaccuccucauggcagccuacucuccuugaguguaugaguagccaggguaaggggguaaaaggauaguaagcauagaaaccacuagaaagugggcuuaa
uggaguucuuguggccucagcucaaugcaguuagcugaagaauugaaaaguuuuuguuuggagacguuuauaaacagaaauggaaagcagaguuuucauuaa
auccuuuuaccuuuuuuuuuucuugguaaucccccuaaaauaacaguaugugggauauugaauguuaaagggauauuuuuuucuauuauuuuuauaauuguac
aaaauuaagcaaauguuaaaaguuuuauaugcuuuauuaauguuuucaaaagguauuauacaugugauacauuuuuuaagcuucaguugcuugucuucuggu
acuuucuguuaugggcuuuuggggagccagaagccaaucuacaaucucuuuuuguuugccaggacaugcaauaaaauuuaaaaaauaaauaaaaacuaauua
agaaa

Figure S9. Multiple adenyl-uridine-rich elements (red) are in 3’-UTR sequence of *TP63* mRNA.

Table S1. Antibodies used in the study

**Name**	**Company**	**Catalog Number**
TDP-43	Proteintech	10782-2-AP
IgG	Invitrogen	02-6102
PABPC1	Proteintech	10970-1-AP
HuR	Proteintech	11910-1-AP
TP63	Proteintech	12143-1-AP
GAPDH	Proteintech	10494-1-AP
β-Tubulin	Proteintech	10094-1-AP
β-actin	Proteintech	66009-1-Ig
Lamin B1	Proteintech	66095-1-Ig

Table S2. Primers for qPCR

Name	Primer Sequence
TP63-F	GCCCCTCCTAGTCATTTGAT
TP63-R	ATCCCTCCAACACAACTGCT
TDP-43-F	GGGTAACCGAAGATGAGAACG
TDP-43-R	CTGGGCTGTAACCGTGGAG
β-actin-F	GGCGGCACCACCATGTACCCT
β-actin-R	AGGGGCCGGACTCGTCATACT
PABPC1-F	CACCGGTGTTCCAACTGTTT
PABPC1-R	TGCTAGACCTGGCATTTGCT
HuR-F	CCCTCTGGATGGTGGTGAAC
HuR-R	AAGCGGTTGAGAAAACGCAC
TP63-ChIP-F	AGTCTTAGGCCTCACCCCAGA
TP63-ChIP-R	TGTGACCACAGCCAAGTGAT

Table S3. Sequences of siRNAs and shRNAs

Name	siRNA or shRNA sequence
NC	Sense 5'-UUCUCCGAACGUGUCACGUTT-3'<br>Antisense 5'-ACGUGACACGUUCGGAGAATT-3'
siTP63-1	Sense 5'- UCUCAAUCUUGUUUGUCGCAC-3'<br>Antisense 5'- GCGACAAACAAGAUUGAGAUU-3'
siTP63-2	Sense 5'- UAACAAUGAUUAAAAUUGGAC-3'<br>Antisense 5'- CCAAUUUUAAUCAUUGUUACU-3'
shTDP-43-1	5'- CGGGCAGGTCAAGAAAGATCTTAACTCGAGTTAAGAT CTTTCTTGACCTGCTTTTT-3'
shTDP-43-2	5'-CGGGCAAACTTCCTAATTCTAAGCCTCGAGGCTTAGAA TTAGGAAGTTTGCTTTTT-3'
siTDP-43-1	Sense 5'-GCCGAACCUAAGCACAAUATT-3'<br>Antisense 5'- UAUUGUGCUUAGGUUCGGCTT-3'
siTDP-43-2	Sense 5'- GGCUGGUAGAAGGAAUUCUTT-3'<br>Antisense 5'- AGAAUUCCUUCUACCAGCCTT-3'
siPABPC1-1	Sense 5'-GCAUGAAGAUGCACAGAAAGC-3'<br>Antisense 5'- UUUCUGUGCAUCUUCAUGCCU-3'
siPABPC1-2	Sense 5'- CCAAUGUUUACAUCAAGAAUU-3'<br>Antisense 5'- UUCUUGAUGUAAACAUUGGUG-3'
siHuR-1	Sense 5'- CGAGCUCAGAGGUGAUCAAAG-3'<br>Antisense 5'- UUGAUCACCUCUGAGCUCGGG-3'
siHuR-2	Sense 5'- CAGUUUCAAUGGUCAUAAACC-3'<br>Antisense 5'- UUUAUGACCAUUGAAACUGGU-3'

Table S4. Mass spectrometry of proteins Co-IP by TDP-43 antibody in KYSE-450 cells

Gene Names	Unique peptides	MW [kDa]	Score	LFQ intensity
MYH9	76	226.53	323.31	6337200000
PLEC	60	505.29	323.31	766270000
KRT2	26	65.432	256.32	2038900000
KRT17	18	48.105	216.5	1058000000
MYH10	14	230.16	161.03	256520000
DSP	24	331.77	157.43	237870000
TDP43	12	41.632	151.06	3735700000
SND1	17	102	138.22	385660000
KRT13	2	49.586	134.43	477990000
SYNCRIP	15	50.65	132.96	619900000
KRT8	9	53.704	109.27	269530000
PABPC1	9	65.118	101.82	336050000
KRT16	7	51.267	99.515	119050000
GAPDH	13	27.87	97.32	770040000
IGF2BP1	5	63.48	80.667	76319000
HNRPK	8	51.218	79.899	178500000
PTBP1	7	62.463	74.249	244930000
CSDA	5	31.947	68.67	226770000
HEL-S-30	9	57.936	68.502	199010000
MYL6	9	16.29	61.126	697730000
FXR1	10	67.27	61.02	102780000
RPS4X	9	27.259	60.198	131840000
XRCC6	9	69.842	58.415	104170000
LRRC59	6	34.93	55.543	84823000
HEL-215	7	29.82	53.09	130480000
RPS8	5	21.879	51.256	61358000
HNRNPA1	7	33.155	51.208	108000000
DDX5	3	57.162	50.348	114510000
RPS12	4	14.515	50.195	283610000
G3BP1	6	47.987	50.029	90558000
EIF4G1	7	158.64	48.268	68768000
IL2	5	15.462	46.504	3210000000
XRCC5	4	64.243	45.191	54739000
HNRNPC	7	28.916	42.87	111790000
RPL9	3	20.775	42.685	82436000
ENO1	6	47.327	41.463	70869000
SLC25A5	4	32.852	41.34	182470000
RPS3A	4	22.459	41.048	133300000
IKBIP	5	39.309	40.858	248850000
SFPQ	6	55.469	40.774	247090000

RPS18	4	17.718	37.452	35119000
RPL30	3	12.656	37.321	52717000
DHX9	4	140.96	37.006	63378000
RPL12	4	17.818	36.771	92456000
RPS28	2	7.8409	35.814	68828000
TUBA1C	4	36.648	34.606	36372000
PABPN1	2	32.749	34.538	23933000
RPL13A	4	21.716	34.495	84478000
RPS5	5	22.391	34.13	182610000
RPS14	3	16.159	33.82	74747000
HEL-S-15	3	18.502	32.902	120120000
DKFZp686P17171	5	42.44	32.849	62491000
RPL13	5	24.265	32.494	73775000
ILF2	3	38.91	31.907	69018000
PHB2	4	27.437	31.678	40486000
MAP4	5	245.37	30.149	75055000
RPL10A	5	24.831	29.788	68254000
UPF1	4	123.03	29.184	34840000
HNRNPM	2	77.569	29.123	128100000
RPS11	4	18.431	28.759	85830000
RPL7	4	30.438	28.508	75971000
RPS19	4	16.06	27.48	54945000
SERPINH1	1	8.76	26.838	32158000
RPLP0	4	27.187	26.53	85552000
NONO	2	53.818	25.7	59633000
HNRPR	4	67.86	25.696	79655000
RPS7	3	22.127	25.572	435540000
RPS13	4	17.222	25.309	99304000
RPS15A	3	11.477	25.198	91211000
IGHG1	4	31.983	24.921	3283400000
FAM120A	4	121.89	24.888	32283000
RPS9	4	22.591	24.772	51825000
PRDX1	3	10.676	24.308	64968000
HNRPF	3	45.671	24.032	87341000
ELAVL1 HUR	3	36.091	23.579	44604000
RPS20	3	13.373	23.317	119450000
rps2	3	25.605	23.153	231900000
RPL18	3	18.732	21.876	61349000
RPL17	3	19.586	20.589	47348000
RPL14	2	23.787	20.512	34668000
